# Physical activity and ability to meet different work requirements among adult working men with or without current depressive symptoms

**DOI:** 10.1007/s00420-020-01595-6

**Published:** 2020-10-30

**Authors:** Ville Päivärinne, Marie Thodén, Hannu Kautiainen, Jari Arokoski, Hannu Koponen, Ari Heinonen, Ilkka Kiviranta

**Affiliations:** 1 Department of Orthopaedics and Traumatology, University of Helsinki and Helsinki University Hospital, P.O. Box 63, 00014 Helsinki, Finland; 2 Department of Physical and Rehabilitation Medicine, Helsinki University Hospital and University of Helsinki, Helsinki, Finland; 3 Primary Health Care Unit, Kuopio University Hospital, Kuopio, Finland; 4 Folkhälsan Research Center, Helsinki, Finland; 5 Old Age Psychiatry, Department of Psychiatry, University of Helsinki and Helsinki University Hospital, Psychiatry, Helsinki, Finland; 6 Faculty of Sport and Health Sciences, University of Jyväskylä, Jyväskylä, Finland

**Keywords:** Physical activity, Depressive symptoms, Work ability, Questionnaires

## Abstract

**Purpose:**

To examine the relationship between leisure-time physical activity (LTPA) and ability to meet different work requirements among adult working men with or without current depressive symptoms.

**Methods:**

We measured LTPA with the long version of the International Physical Activity Questionnaire (IPAQ). The Work Ability Index (WAI) and Beck Depression Inventory (BDI) were used to assess the work ability and depression of 921 Finnish employed male volunteers. Participants were divided into three groups according to the WAI for their work requirements: mental (MENT), physical (PHYS), and an equal amount of mental and physical work (BTH).

**Results:**

When adjusted for age, BMI and employment years, there was a significant difference in weekly LTPA between WAI groups {*p* = 0.003, [F (2902) = 5.58]}, but not for depression. It appeared that participants with depressive symptoms scored lower WAI in each group regardless of LTPA. In addition, a linear relationship was found between higher LTPA and WAI in nondepressed workers in the PHYS [*p* = 0.011, *β* = 0.10 (95% CI 0.03–0.18)] and BTH [*p* = 0.027, *β* = 0.19 (95% CI 0.03–0.34)] groups. Among workers with depressive symptoms, similar linearity was found in BTH [*p* = 0.003, *β* = 0.20 (95% CI 0.03–0.55)]. In group-wise comparison, work requirements {*p* = 0.001, [F (2902) = 11.2]} and depressive symptoms {*p* < 0.001, [F (1902) = 177.0]} related with lower WAI.

**Conclusion:**

Depressive symptoms were associated with lower work ability regardless of the job description. Therefore, higher levels of weekly LTPA was linked with better work ability among nondepressed working men. Workers with depressive symptoms in jobs that require extensive mental or physical work might need more than exercise to improve work ability.

**Electronic supplementary material:**

The online version of this article (10.1007/s00420-020-01595-6) contains supplementary material, which is available to authorized users.

## Introduction

The World Health Organization estimates that a total of 320 million people are living with depression. As a result, depressive disorders are globally ranked as the single largest contributor to nonfatal health loss, and mental disorders, in general, are major contributors to the global burden of disease (James et al. 2018; World Health Organization 2017). In Europe, over a third of the total EU population suffers from mental disorders, which is why identifying strategies for improved prevention are viewed as a primary health challenge of the twenty-first century (Wittchen et al. 2011). Symptoms of depression are viewed as core symptoms of low mood or loss of interest, associated with feelings of inadequacy and hopelessness or sleep problems (Nieuwenhuijsen et al. 2014). Physical activity (PA) is associated with a variety of health benefits, such as reduced overall mortality, improved musculoskeletal health, and stress regulation, with reduced risk of obesity, stroke, cardiovascular disease, and cancer. However, the effects of PA on mental health and depression are still inconsistent (Chekroud et al. 2018; Cooney et al. 2013).

Previous systematic reviews have concluded that exercise could be an effective mediator to treat or prevent symptoms of depression (Malhi et al. 2015; Mammen and Faulkner 2013; Schuch et al. 2016a; Teychenne et al. 2008). However, more studies are needed to identify individual antidepressant moderators such as clinical, biological, and psychological effects on exercise (Schuch et al. 2016a). In addition, different life domains moderate the relationship between PA and mental ill-health, whereas promoting leisure-time physical activity (LTPA) may be most beneficial in terms of mental health promotion and prevention (White et al. 2017). Despite evidence that several psychosocial mechanisms explain the effect of PA on mental health, there is still little evidence as to whether these mechanisms play the same role in different PA domains (White et al. 2017). While depressive disorders are report to be associated with decreased health-related quality of life (HRQoL) (McIntyre et al. 2011), our previous study indicated that relatively high LTPA was associated with positive mental and physical aspects of HRQoL, regardless of total PA (Päivärinne et al. 2018). However, few studies include domain specific, multi-dimensional PA assessment with depressive symptoms and relationship to work ability. Work-related factors are known to represent risk factors for depression and ability to work is considered an important aspect of well-being and health status (Lee et al. 2017). In addition, the increase of PA have been shown to improve health status or work ability (van den Berg et al. 2009; Warburton et al. 2006). Therefore, more research is needed to study whether LTPA could be associate with better work ability, particularly among workers with depressive symptoms.

Krogh and colleagues (Krogh et al. 2009) found that strength training decreased the absence time from work, while another study (Kull et al. 2012) indicated that both LTPA and occupational PA related to depressive symptoms; occupational PA was associated with higher depressiveness and LTPA was related to lower depressiveness. In both of these studies, the majority (Krogh et al. 2009) or all of the participants (Kull et al. 2012) were female. Since a wide range of factors, including sex (Schuch et al. 2016a), can potentially moderate the response of people with depressive disorders, caution is advised when generalizing these results to men.

According to a recent review, almost two thirds (57%) of Finnish men work in male-dominated industries in which depression levels are higher than in other workforce groups. Furthermore, men tend to have lower levels of health literacy than women and are less likely to visit their doctor, which could increase the risk of depressive symptoms being unrecognized and untreated among men (Roche et al. 2016). The main risk factors for depression in male-dominated industries are reported as poor health and lifestyles, unsupportive workplace relationships, job overload and job demands (Battams et al. 2014). It is estimated that approximately 35–50% of employees with depression will take short-term disability leave at some point during their job tenure (McIntyre et al. 2011). Therefore, it is important to identify effective strategies for sustaining work ability and preventing factors that may reduce it, regardless of the job description.

The purpose of this study was to examine the relationship between LTPA and work ability among adult working men between 20 and 40 years of age, with or without depressive symptoms, in relation to different work requirements.

## Methods

### Design and participants

This cross-sectional, population-based study was conducted in 2009 from five cohorts [birth years 1969 (*n* = 67), 1974 (*n* = 139), 1979 (*n* = 228), 1984 (*n *= 229), or 1989 (*n* = 258)] of Finnish men, wherein initially 921 male participants were extracted as a population sample from those who had performed, withdrawn from, or discontinued military service, or had performed an alternative nonmilitary service. Immigrants, people serving a prison sentence, people with mental disorders, and unemployed people were excluded from the study. Informed consent was obtained from all individual participants included in the study.

### Questionnaire

A questionnaire was applied to record PA, work ability, depressive symptoms, economic situation, health behavioral and functional capacity, musculoskeletal disorders, mental disorders, pain, and alcohol consumption. The questions that we used in our study were partly pre-existing questions that were used on the Finnish Health 2000 study (see supplementary material). In addition, the International Physical Activity Questionnaire (IPAQ-long), Work Ability Index (WAI) and Beck Depression Inventory (BDI) questionnaires were used (Beck et al. 1961; Aromaa and Koskinen 2004; International Physical Activity Questionnaire team 2005; Rautio and Michelsen 2014).

### Physical activity

To estimate the level of LTPA we used the long version of the International Physical Activity Questionnaire (IPAQ-long) in the Finnish language, which is known as a valid and reliable instrument for assessing levels and patterns of PA (Hagströmer et al. 2006). In this study, internal consistency was lower [0.18 (95% CI 0.11–0.24)] because the subdomains of PA distributed differently between participants, meaning, some of the participants scored high in LTPA vs. others with high occupational PA but low LTPA. Therefore, there was no correlation between the subdomains of PA. The questionnaire inquires the time participants spent being physically active in the last seven days during leisure time with three levels of intensity (walking, moderate, or vigorous). IPAQ has also been culturally adapted for the Finnish population (Craig et al. 2003). More specific calculations and assessments of the IPAQ have been published previously (Päivärinne et al. 2018). Participants were divided into tertiles based on their metabolic equivalent of task (MET) of total LTPA: I (< 8 MET h/week), II (8–28 MET h/week), and III (> 28 MET h/week).

### Work ability

Participant workability was assessed by the Work Ability Index (WAI), which is a self-reported instrument that assesses work ability and diagnoses, symptoms, and sickness absence to measure health and functional capacity via a single dimension of work ability (Ilmarinen et al. 2005). The WAI consists of seven items regarding both the physical and psychological aspects of work ability. Scores range from 7 (lowest) to 49 (highest). The points of the WAI form the basis for determining the level of work ability according to the following scales: 7–27 (“poor”), 28–36 (“moderate”), 37–43 (“good”) and 44–49 (“excellent”) (Ilmarinen et al. 2005). The WAI has been demonstrated to be a valid instrument for assessing work ability (Lundin et al. 2017). In our study, the internal consistency was 0.75 (95% CI 0.71–0.79).

Participants were divided into three groups depending on their work requirements as described in their responses to the following question: “Are the demands of your work mainly (i.e. more than half of the working time)…” with the response options of 1. Mental work (MENT) 2. Physical work (PHYS) or 3. An equal amount of mental and physical work (BTH).

### Depressive symptoms

We used questions 1 and 4 of the Beck Depression Inventory (BDI) (Beck et al. 1961) to assess participants’ occurrence of depressive symptoms with a cut-off point of ≥ 1. Question 1 proposed the following answer options, with scoring in brackets: “I do not feel sad (0), I feel sad (1), I am sad all the time and I can’t snap out of it (2), I am so sad and unhappy that I can’t stand it (3). Question 4 included the following options: “I get as much satisfaction out of things as I used to (0), I don’t enjoy things the way I used to (1), I don’t get real satisfaction out of anything anymore (2), I am dissatisfied or bored with everything (3).” BDI questions 1 and 4 measure the essential diagnostic criteria of depression, that is, depressive mood and loss of pleasure (American Psychiatric Association 2000).

### Other variables

We also used WAI to assess the participants’ occupational status and diagnosed disorders (Ilmarinen et al. 2005). When assessing alcohol consumption, daily consumption and frequency (weekly/monthly) over the past 12 months were estimated. According to the Finnish guidelines, high-risk alcohol consumption levels for healthy adult males are considered as more than six drinks at once and 23–24 drinks per week (Kauhanen et al. 1992). The numeric rating scale (NRS), which is a reliable and valid instrument for assessing pain (Hawker et al. 2011), was used to assess general pain, neck pain, upper limb pain, lower back pain, and lower limb pain.

### Statistical analyses

Data are presented as means with standard deviation (SD), and as counts with percentages. Statistical comparisons between the groups were made using the analysis of variance (ANOVA) or Chi-squared test. The relationships between work requirements and LTPA or work ability were evaluated using two-way ANOVA. Models included age, BMI and employment years as covariates. The bootstrap method was used when the theoretical distribution of the test statistics was unknown or in the case of violation of the assumptions (e.g. non-normality). Internal consistency were estimated by calculating Cronbach’s alpha with bias-corrected bootstrap 95 per cent confidence intervals. The normality of variables was evaluated graphically and using the Shapiro–Wilk test. Stata 15.1 (StataCorp LP, College Station, TX, USA) was used for the analysis.

## Results

### Participant demographics

The sample consisted of 921 male participants who were engaged in casual or permanent employment. Demographic and clinical characteristics of the study participants classified by work requirements are shown in Table 1. There was a significant difference according to the participants’ age, meaning that older participants were engaged in work that included mainly mental demands. In addition, the groups differed significantly in terms of working-class status, employment years, cardiovascular diseases, mental disorders, alcohol consumption, general pain, lower back pain, lower limb pain, upper limb pain, sick leave days, sufficient funds, work ability, and sitting time. There was also a statistically significant difference (*p* < 0.001) between the participants’ mean scores on BDI questions 1 or 4 of ≥ 1 (10.7 ± 6.7) or < 1 (1.9 ± 2.5).

**Table 1:** Demographic and clinical characteristics of the participants (*N *= 921) according to their work requirements

	Work requirements			p*
	MENT (*N* = 488)	PHYS (*N* = 217)	BTH (*N* = 216)	
Age, mean (SD)	34 (5)	31 (7)	32 (7)	< 0.001
BMI, mean (SD)	26.0 (3.4)	26.1 (4.2)	26.5 (4.3)	0.17
Blue collar, *n* (%)	211 (43)	209 (93)	174 (81)	< 0.001
Employment years, mean (SD)	6.4 (5.1)	7.2 (6.4)	7.7 (6.3)	0.016
Disorders, *n* (%)				
Accidents	44 (9)	27 (12)	28 (13)	0.19
Musculoskeletal disorders	84 (17)	46 (21)	53 (25)	0.069
Cardiovascular disorders	18 (4)	3 (1)	15 (7)	0.011
Lung disorders	21 (4)	8 (4)	10 (5)	0.88
Mental disorders	11 (2)	8 (4)	15 (7)	0.010
Weekly alcohol consumption, mean (SD)	6.0 (6.6)	6.8 (9.8)	8.6 (11.4)	0.006
Pain, NRS^a^, mean (SD)	1.32 (1.76)	2.05 (2.18)	1.83 (2.05)	< 0.001
Lower back pain	1.30 (1.82)	2.10 (2.45)	1.84 (2.14)	< 0.001
Lower limb pain	0.69 (1.34)	1.19 (1.91)	1.07 (1.80)	< 0.001
Neck pain	1.46 (1.93)	1.75 (2.19)	1.82 (2.11)	0.067
Upper limb pain	0.49 (1.20)	1.03 (1.96)	1.13 (2.02)	< 0.001
Sick leave days > 9	51 (10)	48 (22)	42 (19)	< 0.001
Beck Depression Index (BDI), mean (SD)	3.8 (5.0)	3.7 (5.4)	4.7 (6.2)	0.11
BDI question 1 or 4 ≥ 1, *n* (%)	105 (22)	48 (22)	63 (29)	0.076
Sufficient funds, *n* (%)	393 (81)	154 (71)	152 (70)	0.002
Work ability, NRS^a^, mean (SD)	8.9 (1.1)	8.7 (1.4)	8.6 (1.2)	0.002
Sitting time, mean (SD)	52 (18)	35 (20)	39 (22)	< 0.001

^a^*NRS* numeric rating scale

*p < 0.05

**Fig. 1:**
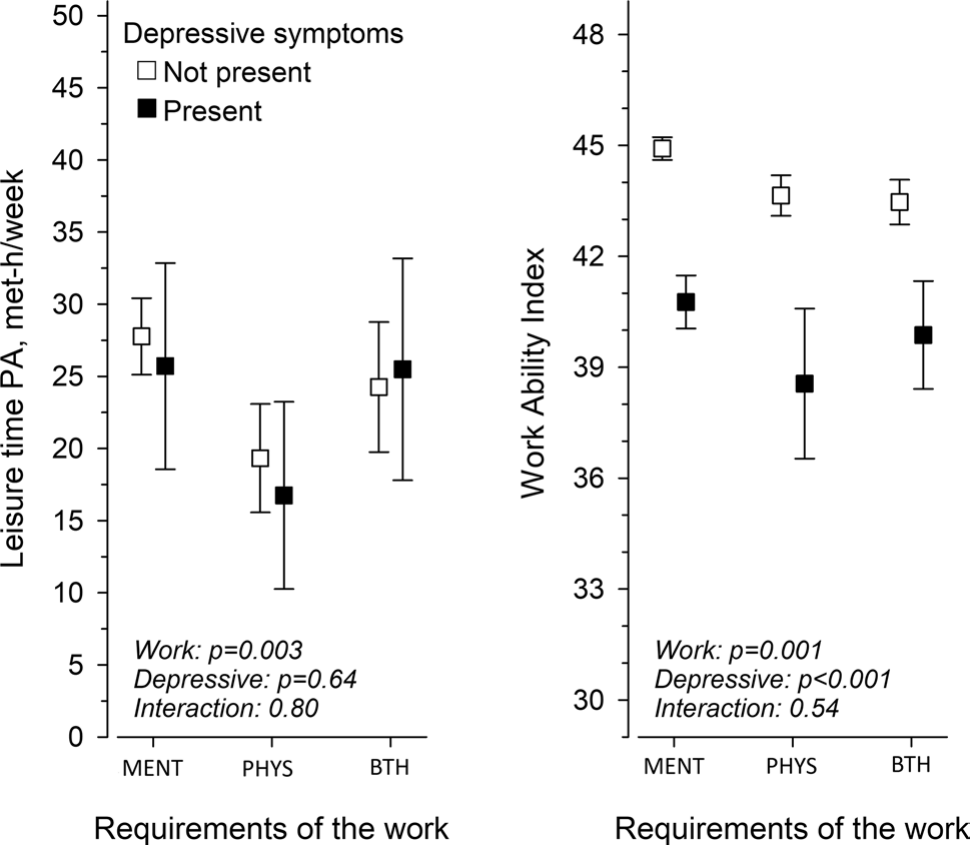
Leisure-time physical activity (LTPA) and Work Ability Index (WAI) in terms of work requirements (adjusted for age, BMI and employment years) and depressive symptoms. Percentages with 95% confidence intervals with (black square) or without (open square) depressive symptoms are shown. *MENT* Mental work, *PHYS* physical work, *BTH* an equal amount of mental and physical work

In Fig. 1, adjusted mean of LTPA (MET h/week) and WAI are shown in terms of work requirements and depressive symptoms. When adjusted for age, BMI and employment years, level of LTPA related significantly to work requirements {*p* = 0.003, [F (2902) = 5.58]}, whereas depressive symptoms were parallel between the groups (interaction *p* = 0.80). On the contrary, in WAI, work requirements related with work ability {*p* = 0.001, [F (2902) = 11.2]} and depressive symptoms with lower WAI {*p* < 0.001, [F (1902) = 177.0]}. Between pairwise group comparisons, in LTPA, there was a significant difference between MENT and PHYS (*p* = 0.005) and PHYS and BTH (*p* = 0.044). In WAI, there was a significant difference between MENT and PHYS (*p* = 0.009) as well as MENT and BTH (*p* = 0.024).

**Fig. 2:**
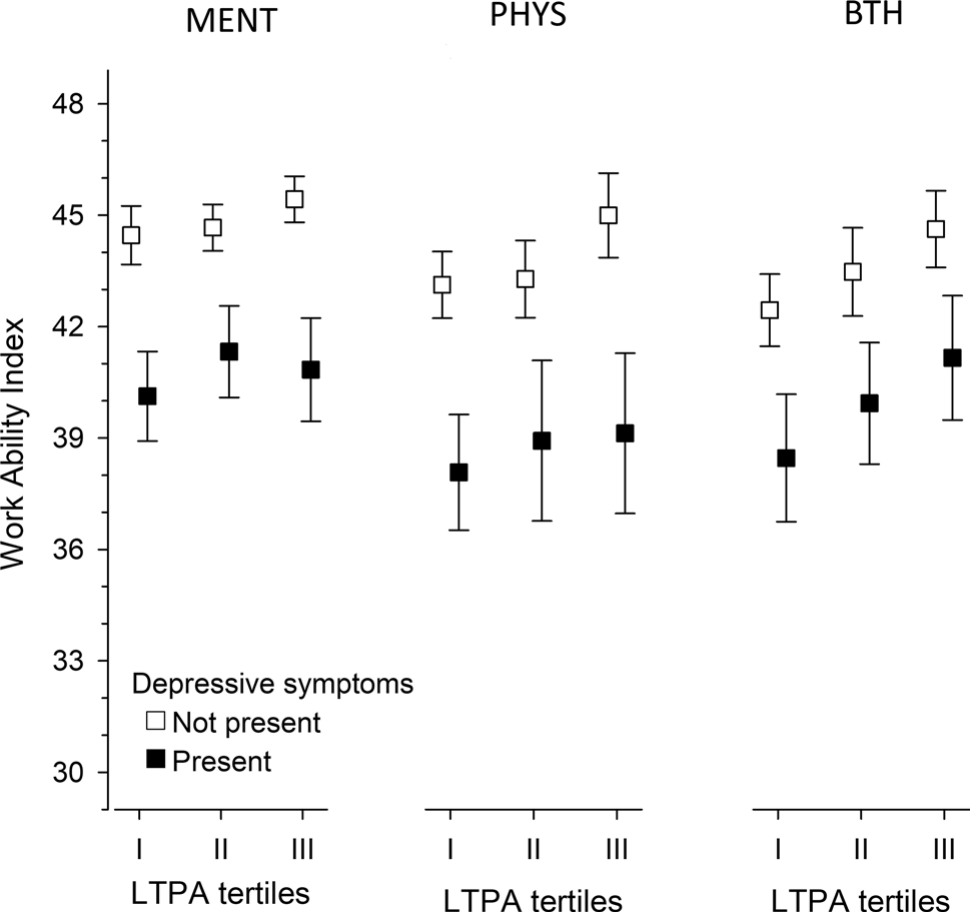
Relationships between the leisure-time physical activity tertiles and depressive symptoms in terms of work requirements (adjusted for age, BMI, and employment years). *MENT* mental work, *PHYS* physical work, *BTH* an equal amount of mental and physical work

As illustrated in Fig. 2, participants with depressive symptoms scored a lower adjusted mean on the WAI regardless of the job description. In addition, a linear association was found between higher LTPA and WAI in nondepressed groups of PHYS [*p* = 0.011, *β* = 0.10 (95% CI 0.03–0.18)] and BTH [*p* = 0.027, *β* = 0.19 (95% CI 0.03–0.34)]. Among workers with depressive symptoms, similar linearity was found in BTH [*p* = 0.003, *β* = 0.20 (95% CI 0.03–0.55)]. This was independent of age, BMI, and employment years.

## Discussion

Our results demonstrate that male workers with depressive symptoms had significantly lower work ability than those without depressive symptoms, regardless of their job demands. Our results indicate a stronger association between higher self-reported LTPA (MET h/week) and work ability among participants without depressive symptoms who engaged in physical labor or had an equal amount of physical and mental aspects in their job description. Among workers with depressive symptoms, similar linearity was only found in the group with an equal amount of physical and mental demands. This was independent of age, BMI and employment years.

A recent systematic review found that male-dominated blue-collar occupations had significantly higher levels of depression than the comparator population. Risk factors for mental illness that are commonly found in these types of occupations usually include poor physical conditions, excessive workloads, monotonous tasks, isolated work, and lack of control (Roche et al. 2016). Moreover, a previous study by Asztalos and colleagues (Asztalos et al. 2012) found that participating in sports was related to less distress in adults aged 20–42 with blue-collar jobs. Furthermore, higher levels of occupational PA have reported to increase the odds of depression (McKercher et al. 2009). Conversely, a review by Lusa and colleagues (Lusa et al. 2020) summarized that workplace PA increased work ability among sedentary workers. In addition, sedentary work has been associated with a higher risk of developing depressive symptoms, while higher levels of LTPA decreased the symptoms (Kuwahara et al. 2015). Even though there is evidence of PA and depression in different job descriptions, as mentioned above, associations between PA in various domains and work ability among workers with or without depressive symptoms remain scarce. Our results emphasize that workers without depressive symptoms benefit from LTPA when measured with WAI. However, workers with depressive symptoms and highly physical or mental job descriptions might need more factors than exercise alone to improve work ability. Therefore, there should not be any restrictive actions against effective lifestyle behaviors such as PA and exercise, which are known to be partially contributed to the risk of developing depression by reducing symptoms, improving HRQoL and health outcomes (Schuch and Stubbs 2019). Instead, the focus should be more about recognizing deleterious contributors among workers with depressive symptoms that possibly hinder the positive interactions between LTPA and work ability. These contributors could be different kind of stressors that vary from one occupation to another, such as exposure to physiological or psychological job strain, noise, high working hours etc. that associate with work-related stress and depressive disorders (Tennant 2001).

A recent study suggested that extreme range of exercise was associated with worse mental health, while team sports and cycling associated with the lowest mental health burden (Chekroud et al. 2018). Participation in sports, a concept of LTPA, could result in the experience of joy and development of mastery and autonomy (Asztalos et al. 2008). In contrast, in highly physical or mental job descriptions, these emotions could be hindered by repetitious work tasks, and thus could be associated negatively with work ability, particularly among workers with depressive symptoms. Moreover, high occupational PA was recently found to be detrimental to health, with increased risk of cardiovascular disorders, sickness absence, and mortality (Coenen et al. 2018).

A previous study has reported a large antidepressant effect of exercise on people with depression (Schuch et al. 2016a). In our study, while there were linear associations between LTPA and WAI in nondepressed groups except MENT, which could be a sign of a ceiling effect (Taylor 2012), a similar association was only found in BTH with depressive symptoms. Therefore, our results show that while there were no significant differences in total LTPA between the groups, the positive association between LTPA and WAI, as it appeared in the nondepressed participants, did not have a similar effect for participants with depressive symptoms. However, the linear association that was found between the BTH with depressive symptoms and LTPA could be explained with the possible variety in the job description of participants with BTH. In other words, the highly monotonous or physically excessive tasks that perhaps appear more among the PHYS and MENT groups do not feature in this particular type of work, or at least not at similar levels. Thus, it does not hinder the interaction of LTPA and work ability and therefore workers cope better.

In our study, participants who were engaged in physical work were also the least physically active outside work, possibly because of the physical demands of their jobs. This is in line with our previous study, wherein participants who recorded the greatest share of their PA from work had the lowest level of LTPA (Päivärinne et al. 2018). In addition, our previous study suggested that workers with higher occupational PA could benefit the most from it, in terms of better work ability (Päivärinne et al. 2019). Moreover, in the present study, LTPA was parallel for the groups with or without depressive symptoms, meaning our results did not indicate that people with depressive symptoms were less likely to engage in physical activity than their counterparts. Similar results are supported by a prospective study by Pinto Pereira and colleagues (Pinto Pereira et al. 2014) wherein higher levels of depressive symptoms predicted less frequent PA only in participants in their early twenties and the association diminished with increasing age. However, workers with depressive symptoms had significantly lower work ability in each of the groups, indicating that depressive symptoms associated with limited work ability. Similar associations have been reported previously for an older Finnish population (Gould et al. 2008).

To detect deteriorating work ability as early as possible, it would be important to detect possible negative associations that could decrease work efficacy and coping at work. A previous study (Hakanen and Schaufeli 2012) suggested that work engagement benefited general well-being and decreased depressive symptoms unlike burnout, which had a negative impact on depressive symptoms. Our study suggests that LTPA could be a factor associated with better work ability in general. In addition, LTPA could moderate the occurrence of depressive symptoms by preventing mental ill-health, as reported previously (Mammen and Faulkner 2013; White et al. 2017).

Despite the possible positive link between exercise and mental health, about half of people with depression will not experience significant improvements from exercise. One potential explanation for this can be the heterogeneity of depression (Schuch et al. 2016b). In our study, a linear association between LTPA and WAI was found in one group with depressive symptoms (BTH). While these results could give some indication that LTPA is an effective method to improve work ability for some, it could also suggest the importance of preventing mental ill-health and depressive symptoms via PA before they arise. Additionally, major depressive disorder is associated with decreased HRQoL, productivity and increased short- and long-term disability, whereas factors such as social support, work demand stress management and interpersonal relations could relate with prevention and treatment (McIntyre et al. 2011). This is why workplace should offer an opportunity to develop tailored strategies that target specific high-risk industries and occupations (Roche et al. 2016). According to our results, these occupations could be jobs that are physically or mentally demanding. Since exercise has multiple benefits to several domains of mental and physical health, it should be promoted to everyone (Schuch and Stubbs 2019). However, individual, social and environmental factors should be recognized when addressing these. Therefore, our study adds useful information to the body of evidence regarding the relationship between LTPA and work ability among male workers with or without depressive symptoms. However, we recommend a certain amount of caution when interpreting these results to women.

### Strengths and limitations

Our study has several strengths. We used validated and widely employed questionnaires, such as the IPAQ long form, to calculate LTPA, BDI, and WAI. Our data was collected and analyzed from a random, homogeneous sample of younger adult men, which allowed us to make reliable generalizations. We also recruited a relatively large sample of Finnish adult working men between 20 and 40 years of age that have not yet been adequately studied. However, limitations should be taken into consideration. Due to the cross-sectional design of the study, the exposure and outcome are simultaneously assessed, so it is not possible to establish a true cause-and-effect relationship. Second, the subjective method of self-assessing LTPA, BDI, and WAI may have resulted in reporting bias that could affect the outcome. Third, caution is advised when generalizing these results to women. Fourth, we were unaware whether the participants were using any kind of antidepressants.

In conclusion, regardless of the job descriptions, workers with depressive symptoms had a lower work ability than nondepressed workers. While linear associations between LTPA and improved work ability were found, this was not the case for workers with depressive symptoms whose occupations had excessive mental or physical aspects. Our results highlight the importance of LTPA for coping at work among male workers.

## Electronic supplementary material

Below is the link to the electronic supplementary material.

Supplementary file1 (PDF 286 kb)
