# Supplementary material for: Physical activity and ability to meet different work requirements among adult working men with or without current depressive symptoms
Source: Int Arch Occup Environ Health. 2020 Oct 30;94(3):451–8. doi: 10.1007/s00420-020-01595-6 (PMC8032589; doi:10.1007/s00420-020-01595-6)
Supplement: Supplementary file 1 — Supplementary file1 (PDF 286 kb) [file 420_2020_1595_MOESM1_ESM.pdf]

## FUNCTIONAL CAPACITY AND QUALITY OF LIFE

**1. Check the box of the following alternatives the one describing best your health and mobility today.**

1.1 Self-care

1 ☐ I have no problems with washing, dressing or in other self-care.
2 ☐ I have some problems with washing, dressing or in other self-care.
3 ☐ I am unable to wash or dress myself.

1.2 Usual daily activities

1 ☐ I have no problems with performing my main activities (e.g. work, study, housework and/or leisure activities.
2 ☐ I have some problems with performing my usual activities.
3 ☐ I am unable to perform my daily activites alone.

**2. Do you have some chronic illness, defect or injury?**

0 ☐ No → go to question number 4
1 ☐ Yes

**3. Estimate how much symptoms, diseases or injuries affect your daily life by checking the box of the most suitable alternative. 0 means for no hindrance at all 10 means for worst possible, i.e. a very significant hindrance.**

3.1 During leisure time activites

| ☐ | ☐ | ☐ | ☐ | ☐ | ☐ | ☐ | ☐ | ☐ | ☐ | ☐ |
|---|---|---|---|---|---|---|---|---|---|---|
| 0 | 1 | 2 | 3 | 4 | 5 | 6 | 7 | 8 | 9 | 10 |

3.2 In household choires

| ☐ | ☐ | ☐ | ☐ | ☐ | ☐ | ☐ | ☐ | ☐ | ☐ | ☐ |
|---|---|---|---|---|---|---|---|---|---|---|
| 0 | 1 | 2 | 3 | 4 | 5 | 6 | 7 | 8 | 9 | 10 |

3.3 At work

| ☐ | ☐ | ☐ | ☐ | ☐ | ☐ | ☐ | ☐ | ☐ | ☐ | ☐ |
|---|---|---|---|---|---|---|---|---|---|---|
| 0 | 1 | 2 | 3 | 4 | 5 | 6 | 7 | 8 | 9 | 10 |

## INCOME AND SICKNESS EXPENDITURE

**4. How would you describe the current balance between income and expenditure in your household?**

1 ☐ We have more than enough money to cover our needs.
2 ☐ There is enough money to cover our needs.
3 ☐ We have to some extent to compromise when deciding what to do with the money.
4 ☐ We have to compromise considerably in our consumption but we can manage with our income.
5 ☐ We have to make major compromises in our consumption and despite of that we do not manage with our income.
6 ☐ I cannot say / it is hard to estimate.

**5. Have your own or your family's sickness expenses been so big this year that you have been forced to…?** (You may choose several options)

1 ☐ Cut down other regular expenses
2 ☐ Use your savings
3 ☐ Take a loan
4 ☐ Accept help from friends and family
5 ☐ Turn to municipal subsistence subsidy
6 ☐ Our sickness expenditure has not been so big

## WEIGHT AND HEIGHT

**6. How much do you weigh at present?** ______ kilos

**7. How much did you weigh when you were 18?** ______ kilos

**8. How tall are you?** ______ cm

## USE OF ALCOHOL

**9. How often have you drunk alchoholic drinks during the past 12 months?**

0 ☐ not once
1 ☐ 6 to 7 times a week
2 ☐ 4 to 5 times a week
3 ☐ 2 to 3 times a week
4 ☐ once a week
5 ☐ approximately once a month
6 ☐ less than once a month

**10. How many portions of alcohol a day did you usually drink on the days when you drank them? One portion is equivalent for a small bottle of beer or cider or 4 cl of spirits or other strong alcohol or 16 cl of wine**

1 ☐ 15 portions or more, how many? _____
2 ☐ 10 to 14 portions
3 ☐ 6 to 9 portions
4 ☐ 3 to 5 portions
5 ☐ less than 3 portions

### TREATMENT OF DRINKING PROBLEMS

**11. During the past 12 months have you used any health or social services due to drinking problems?**

0 ☐ No
1 ☐ Yes

**12. If you answered Yes to the question 11, who has treated you due to your drinking problem?**

1 ☐ Psychiatrist
2 ☐ Other doctor
3 ☐ A nurse or a public-health nurse
4 ☐ Other professional person
